# Case Report: A case of reversible pulmonary bullae induced by camrelizumab: a new immune-related adverse event

**DOI:** 10.3389/fimmu.2025.1619702

**Published:** 2025-09-30

**Authors:** Yun Wang, Shaoshan Wang, Qin Li, Qihao Cui, Jiafu Song, Hong Zheng

**Affiliations:** ^1^ Department of Respiratory and Critical Care Medicine, The First People’s Hospital of Lianyungang, Affiliated Hospital of Xuzhou Medical College, Lianyungang, China; ^2^ Department of Laboratory Medicine, The First People’s Hospital of Lianyungang, Affiliated Hospital of Xuzhou Medical College, Lianyungang, China

**Keywords:** lung squamous cell carcinoma, camrelizumab, immune checkpoint inhibitors, immune-related adverse events, reversible pulmonary bullae

## Abstract

With the widespread clinical application of immune checkpoint inhibitors (ICIs), the treatment of lung squamous cell carcinoma (LUSC) has entered a new era, characterized by equal emphasis on precision medicine and immunotherapy. Among these, programmed cell death protein-1 (PD-1) inhibitors have demonstrated significant efficacy in prolonging patient survival. However, while immunotherapy provides substantial clinical benefits, it may also induce immune-related adverse events (irAEs). We report a case of a 74-year-old male with LUSC who developed reversible pulmonary bullae following camrelizumab treatment. The patient presented with a one-year history of cough. Chest CT revealed a right hilar mass (11×10cm) with pleural effusion. Histopathological analysis of EBUS-TBNA specimens confirmed squamous cell carcinoma. Comprehensive systemic evaluation established the diagnosis of right lung squamous cell carcinoma (cT_4_N_3_M_1a_, stage IVA). The patient received albumin-bound paclitaxel and carboplatin in combination with camrelizumab. During treatment, the patient developed a known immune-related adverse event, interstitial pneumonitis, as well as a previously unreported complication, pulmonary bullae. After discontinuation of camrelizumab and initiation of glucocorticoid therapy (methylprednisolone), the pulmonary bullae showed significant resolution. We believe that the formation of these reversible pulmonary bulla may be associated with two mechanisms. First, immune-mediated airway inflammation and mucus-induced airway obstruction. Second, microvascular or small pulmonary vessel thrombosis leading to localized ischemic injury, which may allow thrombi to enter the airway lumen. Both mechanisms may contribute to a “One-way valve” effect, resulting in alveolar overdistension and bulla formation. This case suggests that pulmonary bullae may represent a rare pulmonary irAE associated with camrelizumab. It provides new clinical insights into immune-related pulmonary complications and offers a valuable reference for the management of similar cases.

## Introduction

Camrelizumab (SHR-1210) is a humanized IgG4κ monoclonal antibody targeting programmed cell death protein-1 (PD-1). It binds to the PD-1 with high affinity, blocking its interaction with PD-L1 and PD-L2. This blockade relieves immunosuppression, restores T-cell immune activity, and enhances the body’s anti-tumour immune response (1–3). Clinical studies have demonstrated that camrelizumab, as an immune checkpoint inhibitor (ICI), shows significant therapeutic efficacy in patients with lung squamous cell carcinoma (LUSC) and has been widely adopted in both first-line and second-line treatment settings (4–7). However, the application of ICIs may trigger various immune-related adverse events (irAEs) due to excessive activation of the immune system (8). Previous studies have reported that common adverse effects of camrelizumab include reactive cutaneous capillary endothelial proliferation (RCCEP), anemia, pyrexia, fatigue, hypothyroidism, proteinuria, and checkpoint inhibitor-related pneumonitis (2, 9, 10). At present, there are no reports indicating that camrelizumab may cause pulmonary bullae. However, we identified a case of reversible pulmonary bullae that was highly related to camrelizumab.

## Case reports

**Figure 1:**
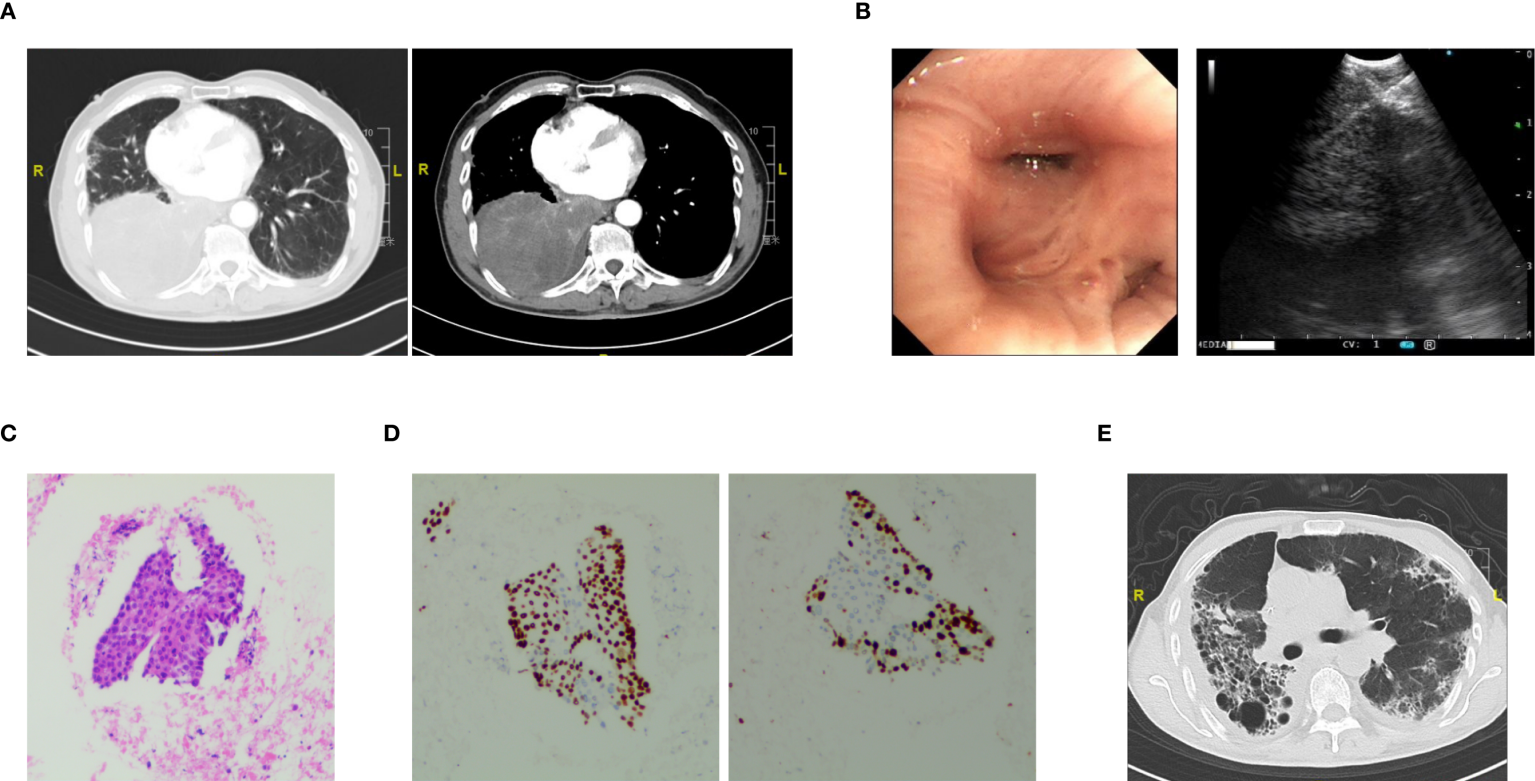
The diagnostic workup on initial visit. **(A)** Contrast-enhanced chest CT showing a right hilar mass with bronchial narrowing and right-sided pleural effusion. **(B)** Bronchoscopic view showing extrinsic compression and obstruction of the right lower lobe bronchus (Left). EBUS image showing a hypoechoic lesion in the right lower lobe with clear boundaries, heterogeneous internal echo, and visible thick blood vessels; fine-needle aspiration was performed while avoiding vascular structures (Right). **(C)** EBUS-TBNA revealing cytological features consistent with squamous cell carcinoma. **(D)** Immunohistochemical staining of biopsy specimens showing strong positivity for P40 (left) and Ki-67 (right). **(E)** Chest CT scan showing bilateral interstitial pneumonitis after four cycles of chemotherapy combined with camrelizumab.

**Figure 2:**
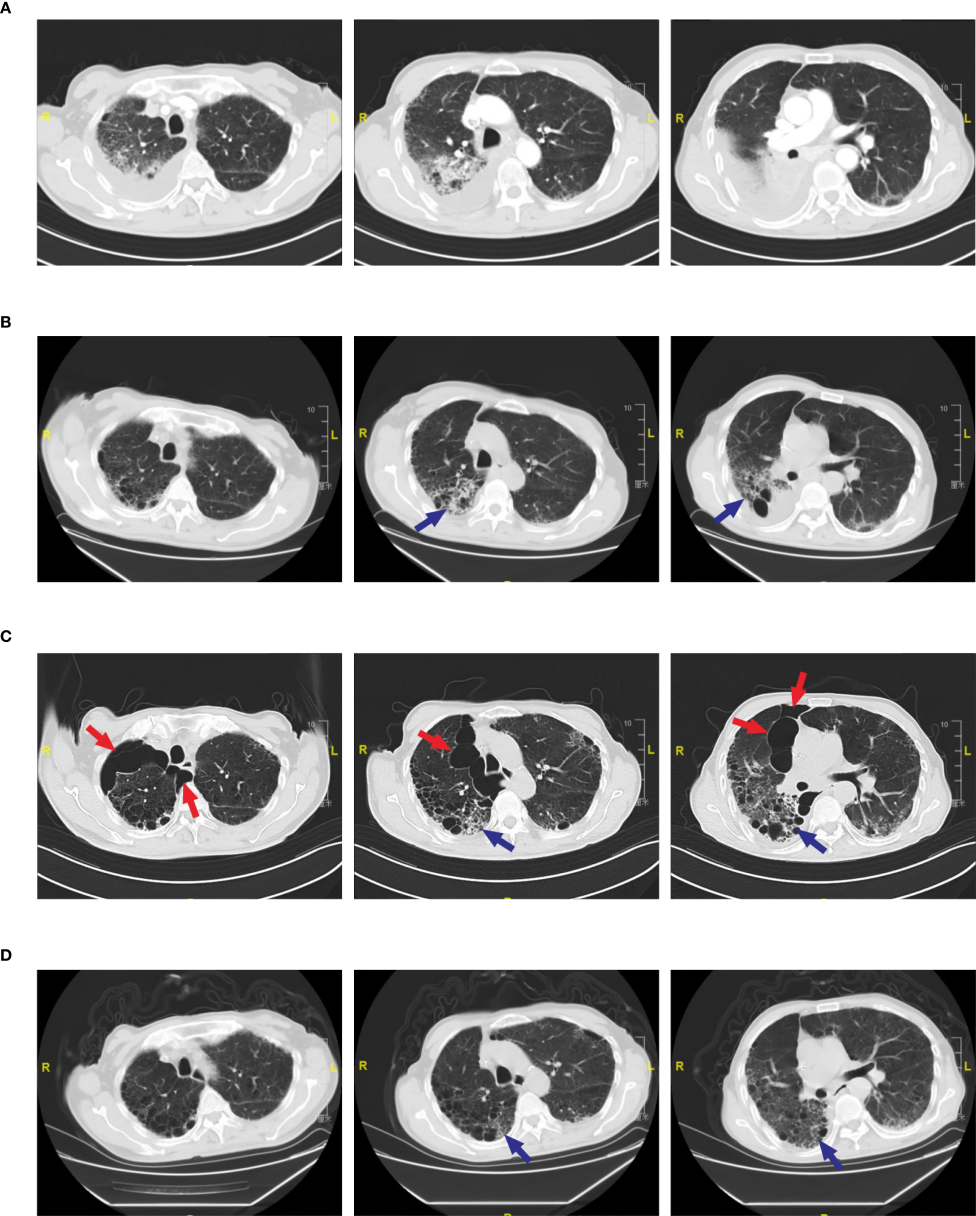
Radiological changes in pulmonary bullae during treatment. **(A)** Pre-treatment CT scan showing no evidence of pulmonary bullae. **(B)** CT scan after two cycles of camrelizumab showing no pulmonary bullae. Blue arrows indicate interstitial pneumonia in the right lung. **(C)** CT scan after four cycles of camrelizumab.(Left) Red arrows indicate multiple pulmonary bullae in the right upper lobe, with the largest measuring 8.75 cm in diameter; (Middle) Red arrows indicate multiple pulmonary bullae in the right upper lobe; blue arrows indicate interstitial pneumonia. (Right) Red arrows indicate multiple pulmonary bullae in the right middle lobe, with the largest measuring 4.85 cm in diameter; blue arrows indicate interstitial pneumonia. **(D)** CT scan following methylprednisolone therapy showing resolution of pulmonary bullae and improvement of interstitial pneumonia. Blue arrow indicates interstitial pneumonia.

**Figure 3:**
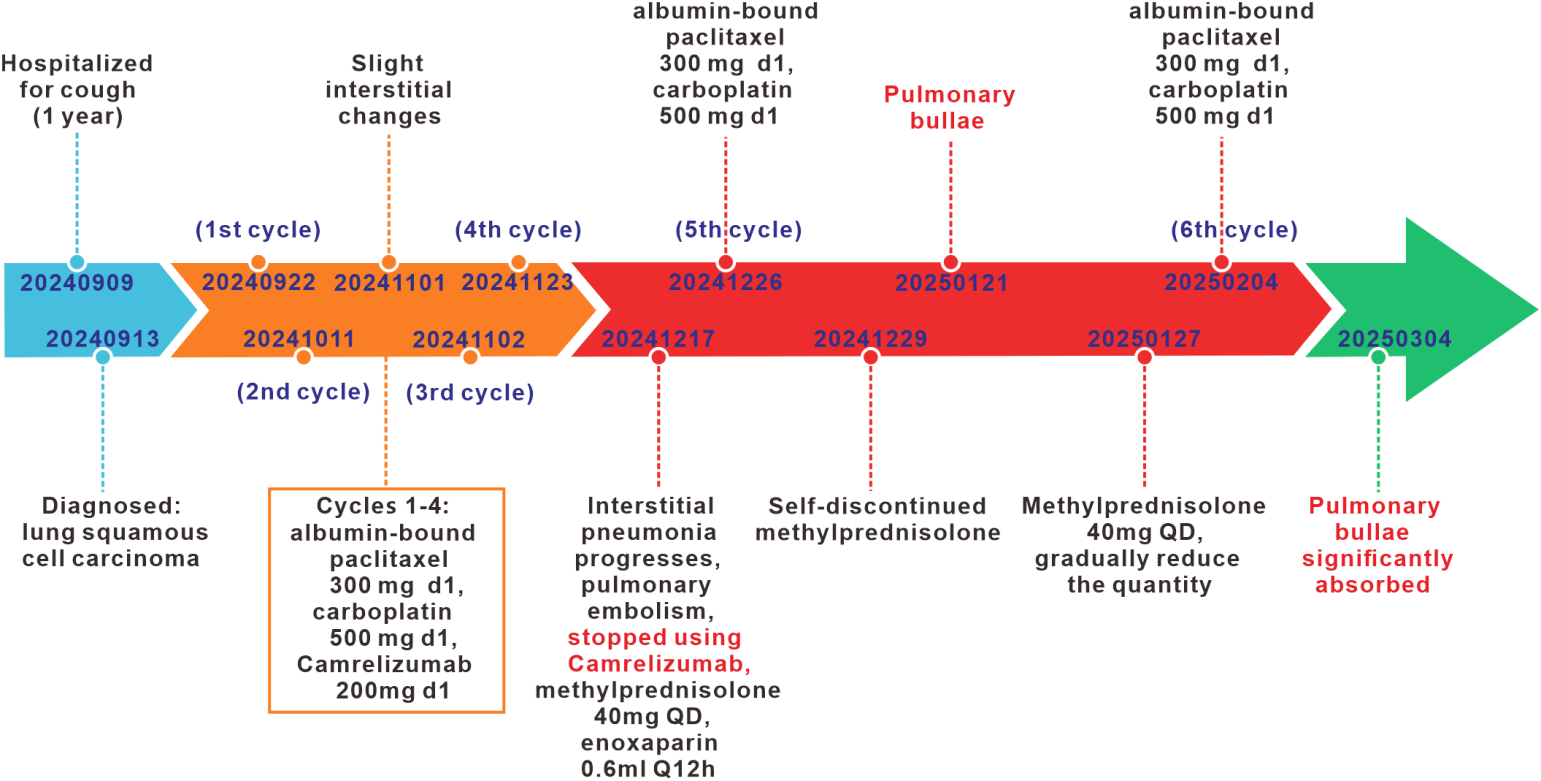
Timeline summarizing the patient’s treatment progression.

A 74-year-old male patient was admitted with a one-year history of persistent cough, without sputum production or shortness of breath. He had a history of smoking (30 pack-years of smoking) but had quit 20 years prior. He denied any family history of lung cancer or other hereditary diseases. Physical examination revealed a clear mental status, no palpable superficial lymphadenopathy, diminished breath sounds in the right lung with audible moist rales. Cardiac auscultation revealed regular rhythm without murmurs in any valvular area. The abdomen was soft and non-tender, with no palpable hepatosplenomegaly. No edema was noted in the lower limbs. Contrast-enhanced chest CT showed a mass (11×10cm) in the right hilar region; localized bronchial narrowing and occlusion; marked heterogeneous enhancement on contrast imaging; enlarged mediastinal lymph nodes; inflammatory changes in the right lung; and a right-sided pleural effusion (**Figure 1A**). Serum tumour markers revealed elevated cytokeratin 19 fragment (39.10 ng/ml) and CEA (12.60 ng/ml). Bronchoscopy revealed external compression and obstruction of the right lower lobe bronchus. EBUS revealed an abnormal echogenic lesion in the right lower lobe. The lesion had well-defined margins, a heterogeneous internal echotexture, and clearly visible vascular structures. Fine-needle aspiration was performed while carefully avoiding the vascular areas (**Figure 1B**). EBUS-TBNA biopsy showed squamous cell carcinoma on histopathological examination (**Figure 1C**). Immunohistochemistry results: TTF-1 (−), NapsinA (−), P40 (3+), CK5/6 (3+), Ki67 (10%+), CK7 (+), CD34 (−) (**Figure 1D**). To further determine the disease stage, right-sided thoracic drainage was performed. Cytology of pleural fluid was negative for malignant cells, but pleural fluid CEA was significantly elevated (128 ng/ml), raising strong suspicion of pleural metastasis. Abdominal contrast-enhanced CT revealed a left renal cyst, prostatic calcification, and a small amount of pelvic effusion. Brain MRI indicated mild lacunar infarction. Cervical and supraclavicular ultrasound showed no significant lymphadenopathy. Whole-body bone scintigraphy and rib MRI revealed no evidence of bone metastases. Based on the AJCC 9th edition TNM staging system, the patient was diagnosed with stage IVA right lung squamous cell carcinoma (cT4N3M1a) (**Figure 2A**). Following multidisciplinary team (MDT) consultation, the patient received two cycles of chemotherapy with albumin-bound paclitaxel (0.3 g on day 1) and carboplatin (500 mg on day 1), administered every 3 weeks, combined with camrelizumab (200 mg on day 1, Q3W). According to the Response Evaluation Criteria in Solid Tumors (RECIST) version 1.1, follow-up imaging indicated partial remission, and CT scans revealed mild interstitial changes in the right lung (**Figure 2B**). After two additional cycles of the same regimen, the patient developed exertional dyspnea. Laboratory tests revealed an elevated D-dimer level (3626 ng/ml). Computed tomography pulmonary angiography (CTPA) indicated right lower pulmonary artery embolism, as well as interstitial changes in both lungs, which had progressed compared to previous imaging (**Figure 1E**). Immune-related pneumonitis is highly suspected. According to the immune-related adverse event management guidelines, the patient discontinued camrelizumab and was administered methylprednisolone 40 mg QD for anti-inflammatory treatment, along with enoxaparin for anticoagulation and symptomatic treatment. The patient completed the fifth cycle of chemotherapy with albumin-bound paclitaxel (0.3 g on day 1) and carboplatin (500 mg on day 1) and, after discharge, self-discontinued methylprednisolone. Follow-up chest CT revealed multiple pulmonary bullae in the right lung (**Figure 2C**). The development of pulmonary bullae following interstitial pneumonitis suggests a strong likelihood of an immune-related cause. Methylprednisolone 40 mg QD treatment was reinitiated, with gradual tapering (total course approximately 6 weeks). During this period, the sixth cycle of chemotherapy was completed with albumin-bound paclitaxel (0.3 g on day 1) and carboplatin (500 mg on day 1). Follow-up CT showed significant improvement in both interstitial pneumonia and pulmonary bullae (**Figure 2D**) (Timeline summarizing the patient’s treatment progression see **Figure 3**). According to the Naranjo Adverse Drug Reaction Probability Scale (**Table 1**), the reversible pulmonary bullae were deemed “most likely” related to camrelizumab.

**Table 1:** Naranjo’s assessment scale in the evaluation of adverse drug reactions.

Related issues	Results	Score
1. Is there any previous conclusive report on this ADR?	no	0
2. Does the ADR occur after the use of suspicious drugs?	yes	2
3. Does the ADR get remission after drug withdrawal or anti-drug application?	yes	1
4. Is the ADR repeated after the use of the suspected drug again?	unknown	0
5. Is there any other reason that can cause the ADR alone?	no	2
6. Does the ADR recur after placebo?	unknown	0
7. Does the drug reach toxic concentration in blood or other body fluids?	unknown	0
8. Does the ADR aggravate with the increase of dose or alleviate with the decrease of dose?	unknown	0
9. Has the patient ever been exposed to the same or similar drugs and had similar reactions?	no	0
10. Is there any objective evidence to confirm the reaction?	no	0
Total score		5

Naranjo’s score ≥ 9 points: definite, 5–8 points: probable, 1–4 points: possible, ≤ 0 points: doubtful.

## Discussion

Although the incidence of pulmonary irAEs is relatively low, their clinical severity is not to be ignored. With the clinical application of immune checkpoint inhibitors (ICIs), an increasing number of rare pulmonary toxicities have been gradually recognized. These toxicities include interstitial pneumonia, ground glass opacities, eosinophilic pneumonia, pulmonary sarcoidosis, organizing pneumonia (OP), adult respiratory distress syndrome, and lung cavitation (11–14). In the present case, the patient developed interstitial pneumonia and previously unreported pulmonary bullae following camrelizumab treatment. The imaging features, clinical symptoms, and temporal progression suggest that pulmonary bullae may represent a rare form of pulmonary irAE. Notably, a previous report described a patient with metastatic squamous cell carcinoma (SCC) who developed bullae-like changes in the lungs on CT imaging after receiving 12 cycles of the PD-1 inhibitor nivolumab (15). This case provides important support for our findings and suggests that immune checkpoint inhibitors may induce similar pulmonary toxicities.

PD-1/PD-L1 inhibitors enhance anti-tumor immune activity by relieving T cell exhaustion, but this widespread immune activation is not entirely tumor-specific and can also induce inflammatory responses in normal tissues (16, 17). Camrelizumab enhances the immune response of Th1 and Th17 helper T cells by inhibiting the PD-1 pathway. At the same time, it suppresses the function of regulatory T cells (Tregs). This disruption of immune tolerance may lead to excessive local inflammation in the lungs (18). IL-17 and other pro-inflammatory cytokines secreted by Th17 cells can drive the recruitment of neutrophils to the lung tissue. Upon activation, these cells release elastase and other tissue-degrading enzymes, leading to the destruction of the alveolar wall structure (19). Additionally, activated CD8^+^ cytotoxic T lymphocytes may also directly mediate damage to alveolar epithelial cells by releasing perforin and granzymes (20). We believe that the mechanism behind this leads to alveolar wall rupture and the fusion of multiple alveoli, resulting in the formation of irreversible pulmonary bullae. However, in this case, the patient showed significant resolution of pulmonary bullae on imaging after discontinuing camrelizumab and initiating corticosteroid therapy, suggesting that the bullae may be reversible. We further speculate that the reversible pulmonary bullae in this case may not be caused by typical alveolar elastic fiber destruction. Instead, they may be caused by another mechanism: immune checkpoint inhibitors can trigger small airway inflammation, edema, or mucus plugging. These changes may lead to airway obstruction and create a “one-way valve” effect, causing distal alveolar air trapping and hyperinflation. This process can ultimately lead to the formation of pulmonary bullae (21, 22). After PD-1 inhibitors activate T cells, they may cause immune-mediated injury to the pulmonary capillary endothelium, leading to increased vascular permeability and pulmonary edema, which in turn affects pulmonary compliance and ventilation distribution, ultimately inducing regional alveolar overinflation (23). In addition, after the tumor was controlled, the patient developed pulmonary embolism. Anticoagulation therapy was administered, and the treatment regimen was adjusted to discontinue camrelizumab while continuing chemotherapy, resulting in clinical improvement. This suggests that the pulmonary embolism may be related to immune therapy rather than tumor progression. It is possible that immune-mediated small vessel vasculitis or a hypercoagulable state led to microvascular or capillary thrombosis in the lungs, resulting in ventilation/perfusion (V/Q) mismatch. Under conditions of relative hyperventilation, reduced perfusion, and localized ischemic injury, microvascular thrombi may enter the airway lumen and act as a “one-way valve.” This one-way valve mechanism causes distal alveolar overdistension, ultimately leading to the formation of pulmonary bullae (24–26). In summary, the mechanisms outlined above may synergistically contribute to immune-related lung injury, resulting in either irreversible or reversible pulmonary bullae.

We reviewed the patient’s treatment history. The patient has a history of smoking but quit 20 years ago. Prior to treatment, the patient had no long-term respiratory symptoms such as persistent sputum production or dyspnea. Imaging studies showed no evidence of emphysema or pulmonary bullae. Considering that the patient had no chronic obstructive pulmonary disease (COPD), it is therefore presumed that the formation of pulmonary bullae is a new onset. During treatment, the patient developed a fever with a CRP level of 110.27 mg/L. Six respiratory pathogen tests, PCT, sputum smear, sputum culture, and blood culture all showed no abnormalities. The patient received antimicrobial therapy with piperacillin-tazobactam (4.5 g Q12H). However, after 72 hours, the fever persisted and PCT level showed no increase, suggesting that the fever was unlikely to be infection-related. After switching to methylprednisolone treatment, the patient’s body temperature returned to normal. This suggests that the fever was likely due to an inflammatory response related to immunotherapy, and the formation of pulmonary bullae is not considered to be caused by infection. During the subsequent chemotherapy, the patient did not experience any further infectious symptoms, which further excluding infection-related fever and infection as causes of the pulmonary bullae. Although paclitaxel and carboplatin can cause pulmonary toxicity, their primary manifestations are diffuse pulmonary fibrosis or interstitial changes, with bulla formation being rare (27). After the sixth cycle of chemotherapy, a follow-up CT scan showed significant absorption and improvement of the pulmonary bullae. This finding suggests that the formation of pulmonary bullae is unrelated to the chemotherapy. It is noteworthy that although irAEs commonly occur during the early phase of treatment, they may also develop in the later stages or even after discontinuation of therapy (28). In this case, the patient developed interstitial pneumonia after receiving camrelizumab immunotherapy combined with chemotherapy. Camrelizumab was discontinued, and methylprednisolone treatment was initiated. However, the patient self-discontinued methylprednisolone after discharge, and follow-up CT scans revealed multiple pulmonary bullae in the right lung. This suggested progression of immune-related lung injury. After the patient received education, methylprednisolone was resumed according to the prescribed regimen. Follow-up CT scans showed significant absorption of the pulmonary bullae and marked improvement in interstitial pneumonia. These findings further strengthen the causal inference that the pulmonary bullae were immune-related. In conclusion, we propose that pulmonary bullae may represent a rare form of pulmonary irAE.

This case provides important insights into the identification of a new form of pulmonary adverse reaction related to immunotherapy, and also suggests the need for early recognition and prompt management of atypical irAEs in clinical practice. However, as this is a single case observation, a definitive causal relationship cannot be fully established. Further validation through large-scale, multicenter studies is required to elucidate the potential association and underlying mechanisms between immunotherapy and pulmonary bulla formation. Future efforts should focus on advancing mechanistic research and improving radiologic recognition to optimize risk management strategies for immunotherapy, thereby enhancing survival outcomes and quality of life in patients with lung cancer.

## Data Availability

The original contributions presented in the study are included in the article/supplementary material. Further inquiries can be directed to the corresponding authors.
